# Glycogen debranching enzyme (AGL) is a novel regulator of non-small cell lung cancer growth

**DOI:** 10.18632/oncotarget.24676

**Published:** 2018-03-30

**Authors:** Craig S. Richmond, Darby Oldenburg, Garrett Dancik, David R. Meier, Benjamin Weinhaus, Dan Theodorescu, Sunny Guin

**Affiliations:** ^1^ Medical Research, Gundersen Medical Foundation, La Crosse, WI, USA; ^2^ Department of Mathematics and Computer Science, Eastern Connecticut State University, Willimantic, CT, USA; ^3^ Department of Surgery (Urology), University of Colorado, Aurora, CO, USA

**Keywords:** non-small cell lung cancer, hyaluronic acid, AGL, HAS2, RHAMM

## Abstract

Glycogen debranching enzyme (AGL) and Glycogen phosphorylase (PYG) are responsible for glycogen breakdown. We have earlier shown that AGL is a regulator of bladder tumor growth. Here we investigate the role of AGL in non-small cell lung cancers (NSCLC). Short hairpin RNA (shRNA) driven knockdown of AGL resulted in increased anchorage independent and xenograft growth of NSCLC cells. We further establish that an increase in hyaluronic acid (HA) synthesis driven by Hyaluronic Acid Synthase 2 (HAS2) is critical for anchorage independent growth of NSCLC cells with AGL loss. Using gene knockdown approach against HAS2 and by using 4-methylumbelliferone (4MU), an inhibitor of HA synthesis, we show that HA synthesis is critical for growth of NSCLC cells that have lost AGL. We further show NSCLC cells without AGL expression are dependent on RHAMM for HA signaling and growth. Analysis of NSCLC patient datasets established that patients with low AGL/high HAS2 or low AGL/high RHAMM mRNA expression have poor overall survival compared to patients with high AGL/low HAS2 or high AGL/low RHAMM expression. We show for the first time that loss of AGL promotes anchorage independent growth of NSCLC cells. We further show that HAS2 driven HA synthesis and signaling via RHAMM is critical in regulating growth of these cancer cells with AGL loss. Further patients presenting with low AGL and HAS2 or RHAMM over expressing tumors might present the ideal cohort who would respond to inhibitors of HA synthesis and signaling.

## INTRODUCTION

Amylo-alpha-1-6-glucosidase-4-alpha-glucanotransferase (AGL) along with glycogen phosphorylase (PYG) breaks down glycogen in human cells [1]. Loss of AGL is known to cause Glycogen Storage Disease III (GSDIII) which results in accumulation of abnormally branched glycogen predominantly in liver and skeletal muscle [2]. GSDIII symptoms can be resolved by maintaining a diet high in protein [2]. We have recently identified AGL as a suppressor of bladder tumor growth and established for the first time that AGL plays a role in cancer biology [3]. We have shown that loss of AGL results in aggressive anchorage dependent and independent growth of bladder cancer cells [3]. We have also shown that AGL mRNA and protein expression is a predictor of bladder patient outcome [3].

By thorough experimentation we validated that AGL's enzymatic function does not play a role in regulating tumor growth [3]. We also established that inhibition of glycogen breakdown in general, due to loss of AGL, does not promote tumor growth [3]. Through metabolomics and transcriptomic analysis we identified that loss of AGL makes bladder cancer cells dependent on Serine Hydroxymethyltransferase 2 (SHMT2) driven glycine synthesis and Hyaluronic Acid (HA) Synthase 2 (HAS2) driven HA synthesis for aggressive growth [3–5].

Here we investigate the role of AGL in non-small cell lung cancer. We show that loss of AGL promotes aggressive anchorage independent and xenograft growth of NSCLC cells. This is the first report showing AGL as a novel regulator of NSCLC anchorage independent growth. We also illustrate that the AGL low NSCLC cells are vulnerable to inhibition of HAS2 dependent HA synthesis and HA signaling via RHAMM.

## RESULTS

### Loss of AGL promotes anchorage independent and xenograft growth on NSCLC cells

**Figure 1:**
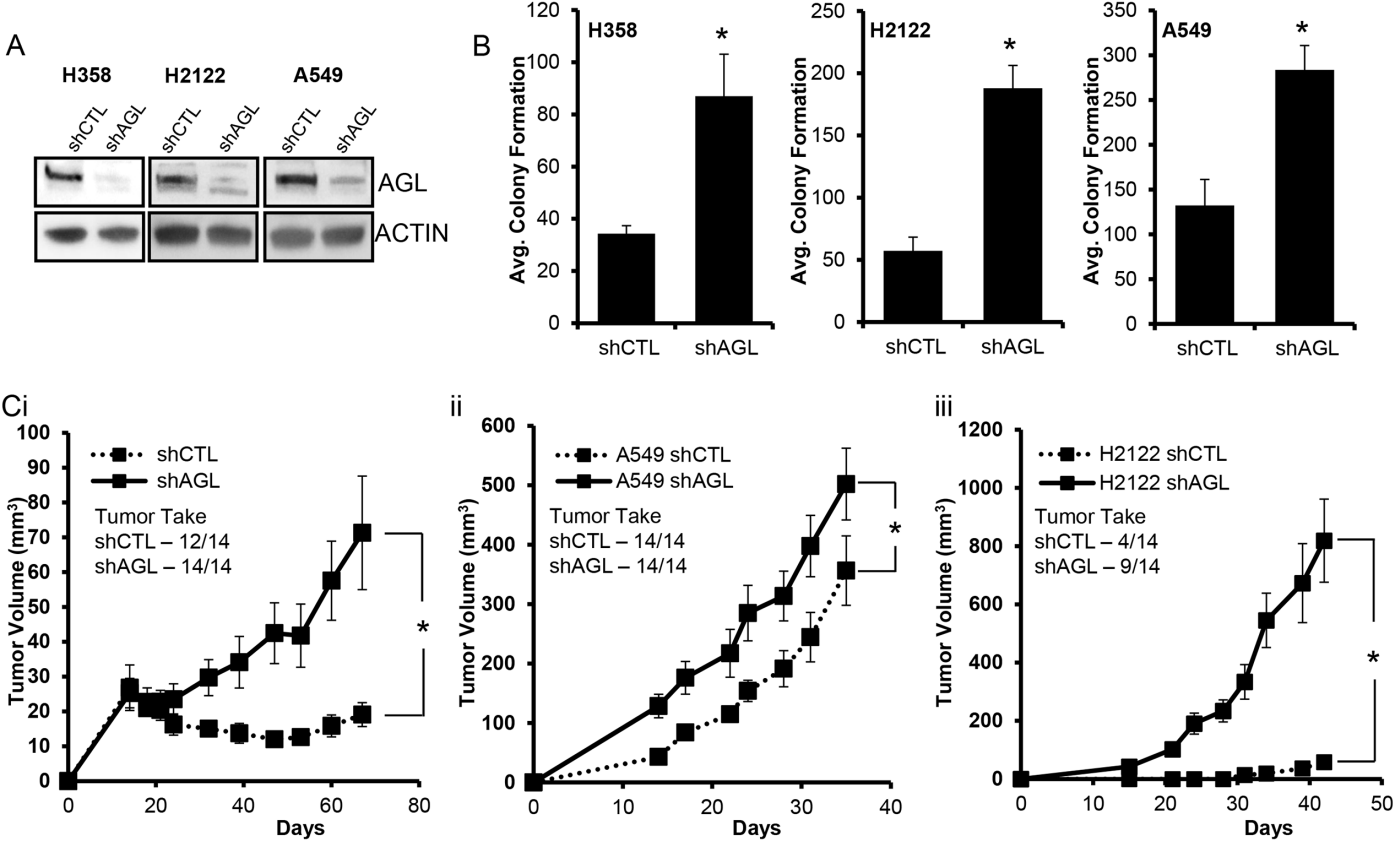
Glycogen Debranching Enzyme Loss and Tumor Growth **(A)** AGL gene knockdown was validated by Western blot in the NSCLC cell lines. Cells transduced with control shRNA (shCTL) and cells transduced with AGL specific shRNA (shAGL). **(B)** Anchorage independent growth (n=3) of NSCLC cells with (shCTL) and without AGL (shAGL) expression. 15×10^3^ cells were plated in 6 well plate for agar growth. Results are shown as mean±SD, ^*^p<0.05 by Student's t-test. **(C)** Xenograft growth of NSCLC cells with (shCTL) and without AGL (shAGL) expression. 7 mice per group were injected in the right and left flank with shCTL and shAGL i) H358 (4×10^6^ cells/site), ii) H2122 (1.0×10^5^ cells/site) or iii) A549 (2×10^6^ cells/site) cells. Tumors were measured as described in Material and Methods. Results are shown as mean±SEM, ^*^p<0.05 by Student's t-test.

To test the hypothesis that loss of AGL impacts the growth of NSCLC cells we selected three well established NSCLC cell lines (H358, H2122, A549) for our study. We carried out AGL knockdown (shAGL) in these cells using the previously described and validated AGL shRNA construct TRCN0000035082 from Sigma-Aldrich [3–5]. Successful knockdown of AGL protein expression in these three cell lines was demonstrated by Western Blot analysis (Figure 1A). Loss of AGL promoted anchorage independent growth of these cell lines as seen in Figure 1B. We used a 2^nd^ shRNA construct against AGL (shAGL') which targeted the 3'UTR region (TRCN0000419324). Loss of AGL using this construct also resulted in increased anchorage independent growth of H2122 and H358 NSCLC cell lines proving that the effect we see on anchorage independent growth is specific to AGL loss (Supplementary Figure 1). However loss of AGL did not result in increased proliferation of NSCLC cells H2122 and H358 in a mono-layer anchorage dependent growth assay (Supplementary Figure 2). Next we investigated the role of AGL knockdown in promoting xenograft growth of these NSCLC cells. We show that loss of AGL promoted rapid xenograft growth of H358, H2122 and A549 cells (Figure 1C), however cells with and without AGL loss had similar tumor take (Figure 1C).

### AGL's role in NSCLC is independent of its role in glycogen metabolism

**Figure 2:**
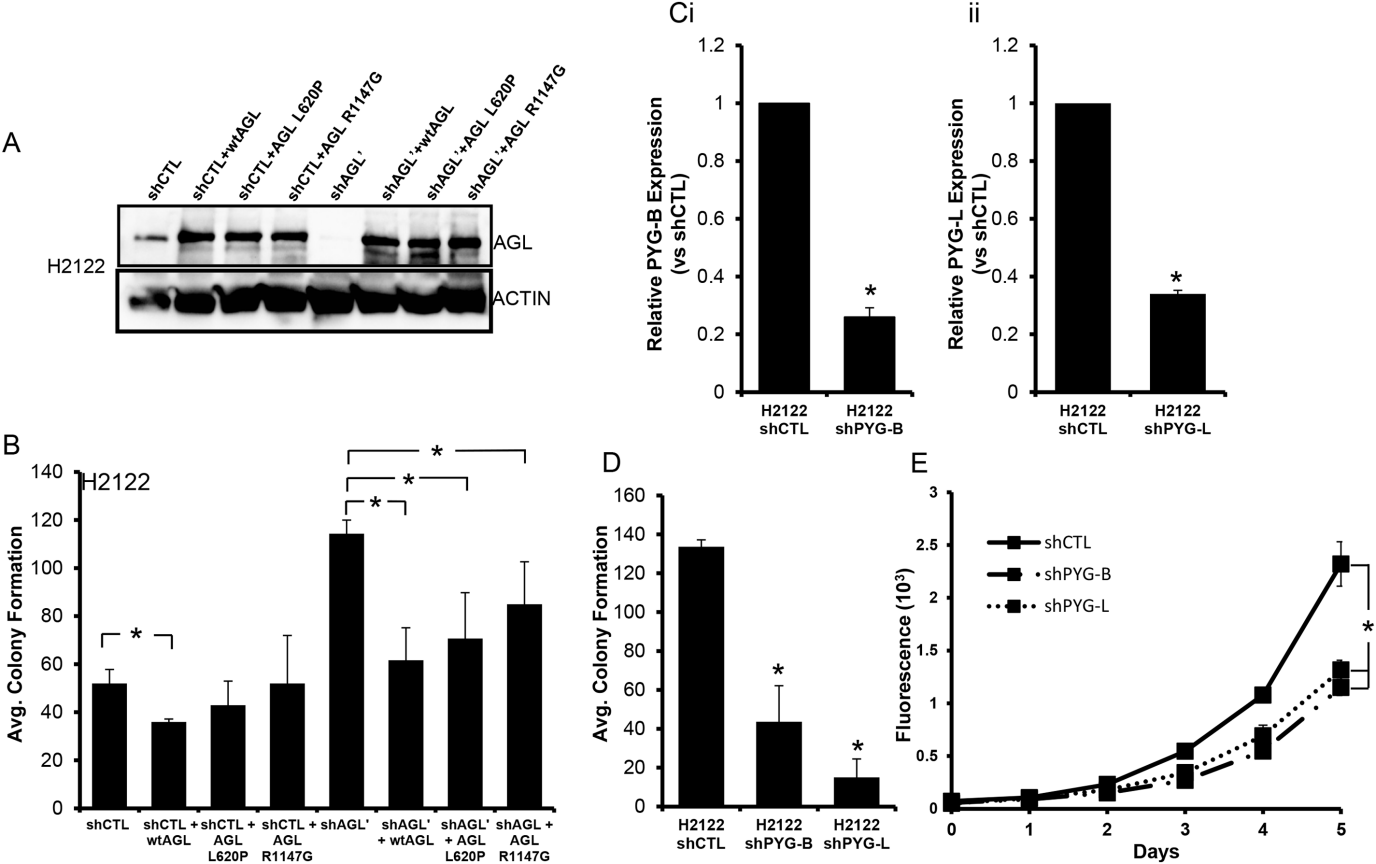
Glycogen Metabolism and Tumor Growth **(A)** AGL expression in H2122 cells transduced with nontarget shRNA (shCTL) and cells transduced with shRNA against AGL specific to 3'UTR region (shAGL') stably overexpressing WT-AGL and enzymatic null AGL. **(B)** Anchorage independent growth (n=3) of H2122 cells with (shCTL) and without AGL (shAGL') expression stably overexpressing WT-AGL and enzymatic null AGL. 15×10^3^ cells per cell type were plated in 6 well plate for soft agar growth. **(Ci-ii)** qRT-PCR demonstrating efficacy of glycogen phosphorylase brain (shPYG-B) and liver (shPYG-L) isoform depletion in H2122 cells stably transduced with shRNA against glycogen phosphorylase brain and liver isoform. **(D-E)** Anchorage independent (n=3) and dependent (n=6) growth of H2122 cells transduced with nontargeted shRNA and shRNA against glycogen phosphorylase liver (shPYG-B) and brain (shPYG-L) isoform. 15×10^3^ and 10^3^ cells were plated in 6 well plates and 96 well plate for monolayer growth and agar growth. Results are shown as mean±SD, ^*^p<0.05 by Student's t-test.

AGL has two known enzymatic functions, glucosidase and transferase [6, 7]. We generated two AGL enzymatic null mutants L620P and R1147G lacking the transferase and glucosidase function respectively [3, 6, 7]. We stably overexpressed AGL and AGL enzymatic null mutants in H2122 and A549 cells with and without AGL knockdown (Figure 2A, Supplementary Figure 3A). H2122 and A549 cells where AGL knockdown was achieved with 3'UTR targeting AGL shRNA (shAGL') were used for exogenous overexpression of AGL (Figure 2A, Supplementary Figure 3A). However in A549 cells we were unable to sufficiently overexpress AGL in the control cells (shCTL) but were able to overexpress the protein in the AGL knockdown cells (shAGL') (Supplementary Figure 3A). As predicted we saw an increase in anchorage independent growth with the loss of AGL expression while the stable overexpression of WT-AGL and AGL enzymatic null mutants reduced anchorage independent growth of the AGL knockdown cells (Figure 2B, Supplementary Figure 3B). This established that AGL's known enzymatic functions do not play a role in tumor biology since both WT and the enzymatic null AGL rescued the increased growth phenotype seen with AGL loss.

Next we knocked down glycogen phosphorylase isoforms (brain and liver) in H2122 and A549 cells (Figure 2C, Supplementary Figure 3C). Glycogen phosphorylase is the rate limiting enzyme in glycogen breakdown [1, 8]. Loss of glycogen phosphorylase isoforms reduced anchorage independent and dependent growth of H2122 cells (Figure 2D, 2E). Loss of glycogen phosphorylase isoforms also inhibited anchorage independent growth of A549 cells (Supplementary Figure 3D). This establishes that inhibition of glycogen breakdown with loss of AGL protein or its enzymatic function does not promote NSCLC growth when AGL is lost.

### Loss of AGL results in hyaluronic acid (HA) synthase 2 (HAS2) driven HA synthesis

**Figure 3:**
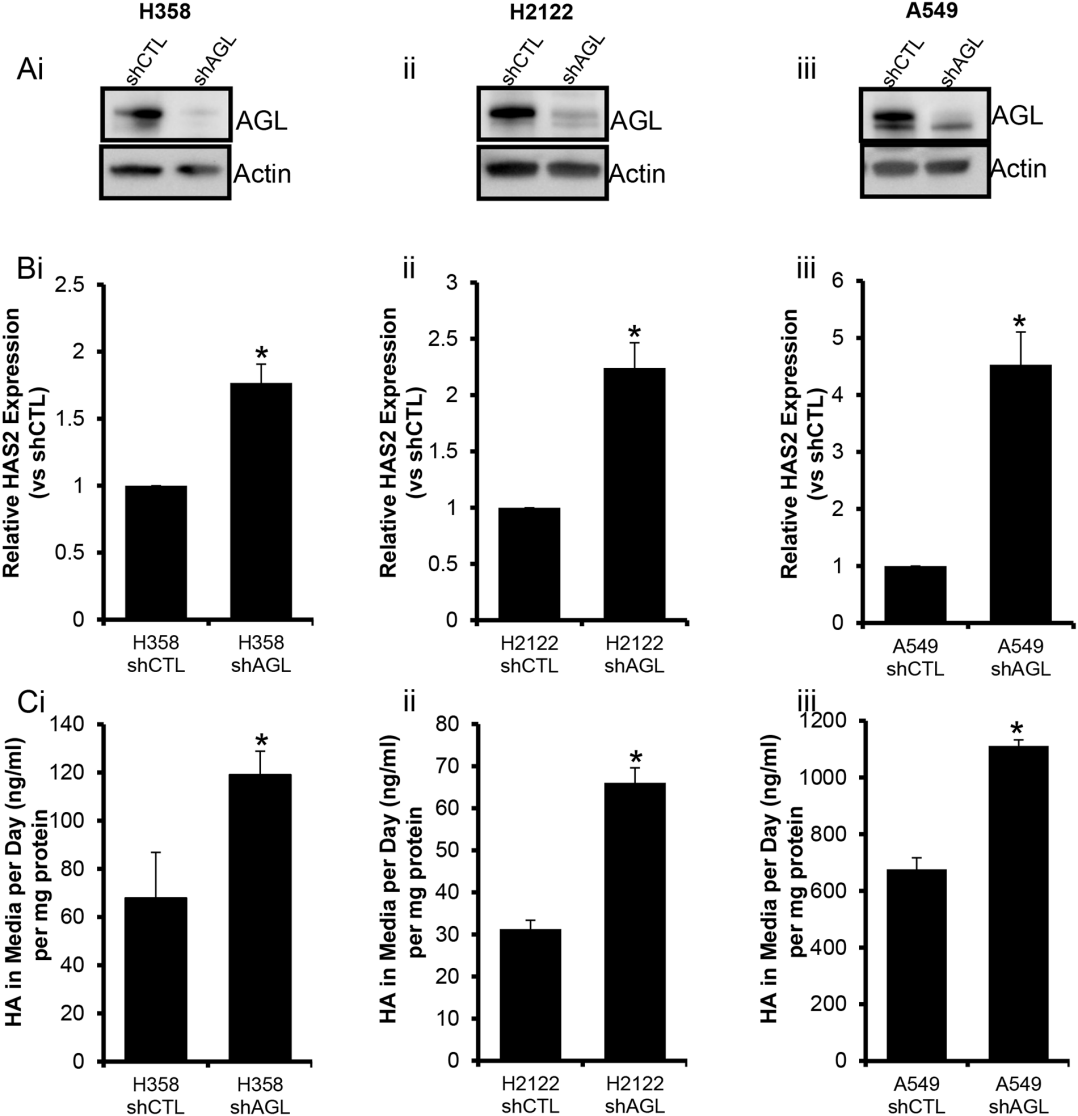
HAS2 Expression and HA synthesis with AGL Loss **(Ai-iii)** AGL gene knockdown was validated by Western blot in the NSCLC cell lines H358, H2122 and A549 respectively. Cells transduced with control shRNA (shCTL) and cells transduced with AGL specific shRNA (shAGL). **(Bi-iii)** qRT-PCR demonstrating the expression of HAS2 in NSCLC cells with and without AGL expression (n=3). **(Ci-iii)** HA secreted into the media by NSCLC cells H358, H2122 and A549 cells with (shCTL) and without AGL (shAGL) expression detected by HA ELISA. Briefly, the cells were plated in 6 welled dish, next day fresh media was added to the cells and HA was measured in the media 24hrs later (n=3). Results are shown as mean±SD, ^*^p<0.05 by Student's t-test.

HAS2 driven HA synthesis is known to promote anchorage independent growth [9, 10]. We have earlier shown that loss of AGL results in increased HAS2 expression and HA synthesis in bladder tumors [4]. Here we test the hypothesis, that HAS2 overexpression and increased HA synthesis is also prevalent in AGL knockdown NSCLC cell lines. We carried out RT-PCR for HAS2 expression as before. We see that with loss of AGL there is an increase in HAS2 expression (Figure 3A, 3B). The increase in HAS2 expression also correlated with increased HA synthesis by the AGL knockdown NSCLC cells compared to cells transduced with control plasmid (Figure 3C). We further investigated expression of the other HAS isoforms HAS1 and HAS3 in the NSCLC cell lines with AGL loss. HAS1 and HAS3 expression was not consistently upregulated or downregulated in the H358 and H2122 cells following knockdown of AGL (Supplementary Figure 4) therefore HAS1 and HAS3 were not investigated further.

### HAS2 driven HA synthesis is important for proliferation for NSCLC cells with AGL loss

**Figure 4:**
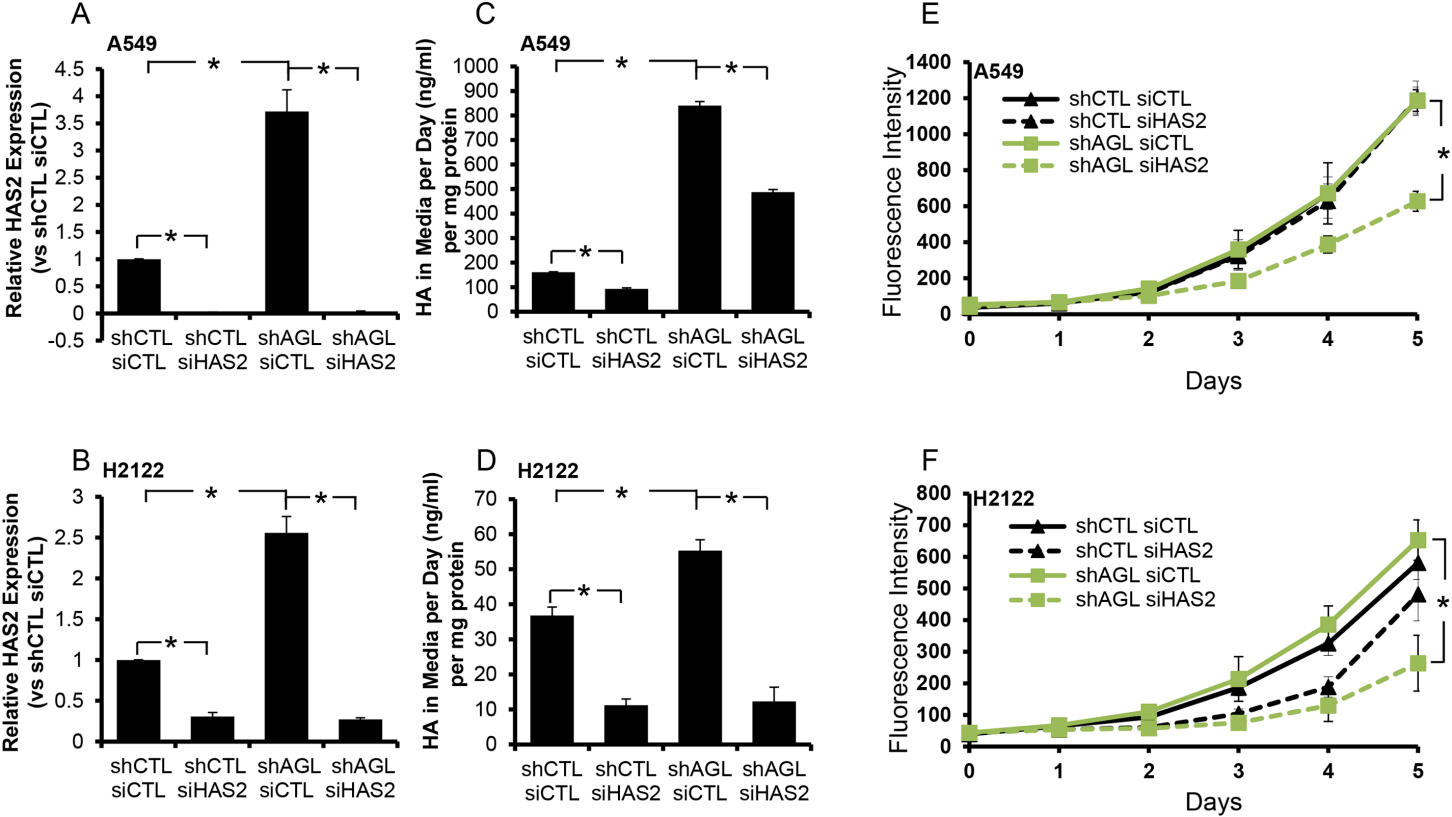
HAS2 loss and growth of NSCLC cells with AGL loss **(A, B)** qRT-PCR demonstrating efficacy of HAS2 depletion in A549 and H2122 control (shCTL) and AGL knockdown (shAGL) cells. Cells were plated and 24hrs later transfected with scrambled (siCTL) or directed siRNA against HAS2 (siHAS2). Details of siRNA used are in Materials and Methods. Cells were harvested at 48hrs for mRNA followed by qRT-PCR analysis (n=3). **(C, D)** HA secreted by A549 and H2122 control (shCTL) and AGL knockdown (shAGL) cells after depletion of HAS2. Cells were plated and 24hrs later transfected with scrambled (siCTL) or directed siRNA against HAS2 (siHAS2). 48 hrs after transfection media was changed on the cells. 24hrs later media was collected for HA ELISA (n=3). **(E, F)** Proliferation of A549 and H2122 control (shCTL) and AGL knockdown (shAGL) cells after depletion of HAS2. Cells were plated and 24hrs later transfected with scrambled (siCTL) or directed siRNA against HAS2 (siHAS2). 48 hrs after transfection cells were plated in 96 welled dish (10^3^ cells/well) (n=6) for proliferation over 5 days. Cell proliferation was measured by CyQUANT assay. Results are shown as mean±SD, ^*^p<0.05 by Student's t-test.

We carried out transient depletion of HAS2 in NSCLC H2122 and A549 cells with and without AGL expression using a siRNA construct which we have previously validated [4, 5]. Using this siRNA we were able to achieve knockdown of HAS2 in both the cell lines +/− AGL expression (Figure 4A, 4B). We saw an increase in HA synthesis with AGL loss in both A549 and H2122 cells (Figure 4C, 4D). In both A549 and H2122 cell lines, control and AGL knockdown, we were able to achieve a significant decrease in HA levels with HAS2 depletion (Figure 4C, 4D).

Next we tested cell proliferation of A549 and H2122 +/− AGL cells after transient transfection with control siRNA and siRNA against HAS2 over 5 days. As we have previously seen (Supplementary Figure 1), loss of AGL did not result in increased proliferation of the NSCLC cell lines. However loss of HAS2 predominantly slowed down the proliferation of the AGL knockdown NSCLC cell lines (Figure 4E, 4F) even though HA synthesis was inhibited in both the control (shCTL) and the AGL knockdown cells (shAGL) with loss of HAS2 (Figure 4C, 4D). We also observed that addition of low molecular weight HA (20μg/ml) to H2122 and A549 cells expressing wild-type AGL promoted their growth (Supplementary Figure 5). This implies that when these NSCLC cells are exposed to high HA level they grow more aggressively. With loss of AGL and increased HAS2 driven HA synthesis these cells are exposed to high HA for prolonged period of time making them more dependent on HA for growth. The NSCLC cells expressing AGL (shCTL) are normally exposed to lower levels of HA hence their growth is not as dependent on HA as seen from their proliferation +/− HAS2 knockdown (Figure 4E, 4F).

### 4-Methylubmelliferone (4MU) reduce HA synthesis and growth of AGL knockdown NSCLC cells

**Figure 5:**
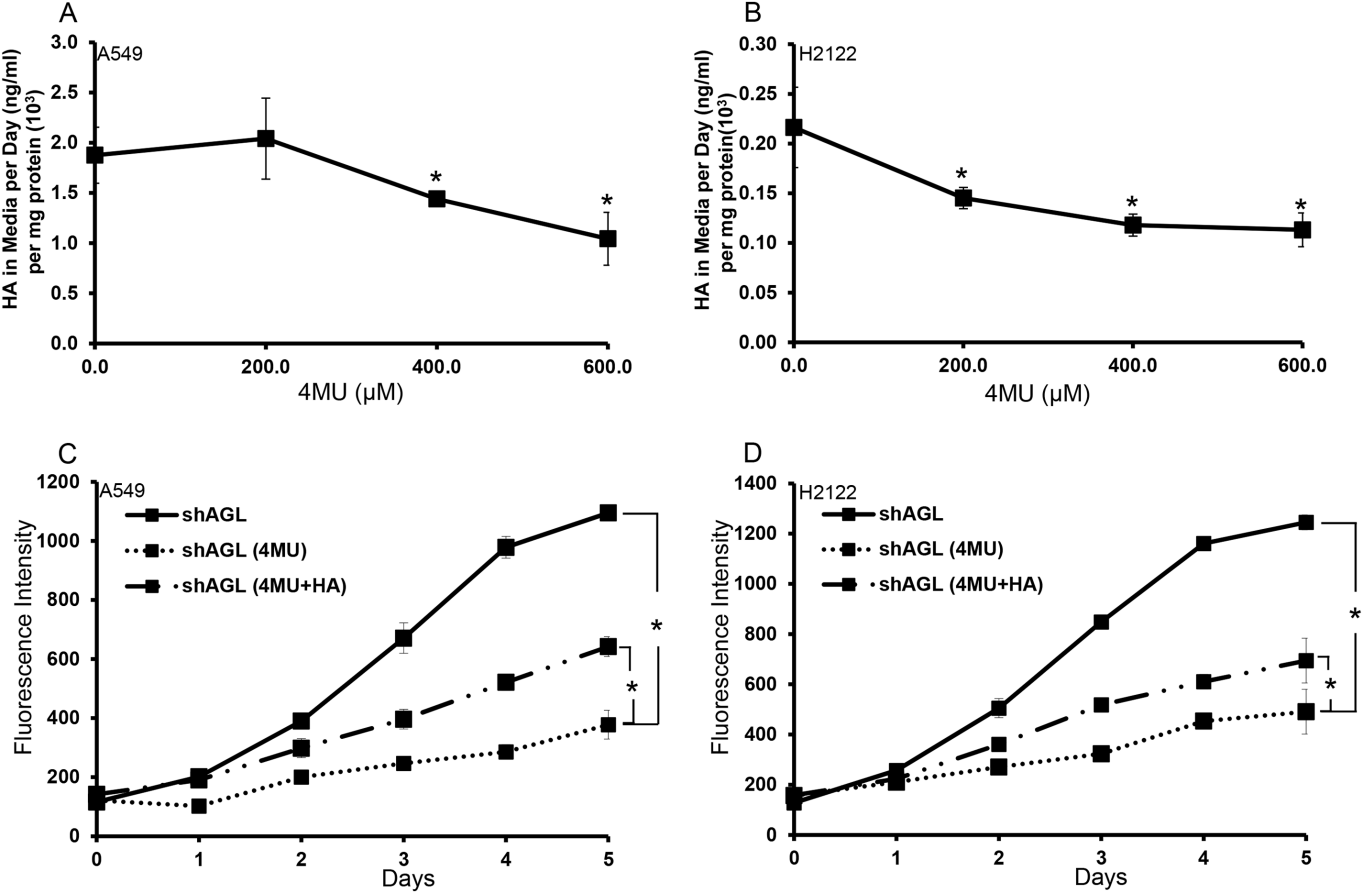
Effect of 4MU on NSCLC cells with AGL loss **(A, B)** A549 and H2122 cells with AGL knockdown (shAGL) were plated in 6 well plates (n=3). 24hrs later media was changed and 4MU was added at different concentrations. HA in the media was measured 24hrs later by ELISA. **(C, D)** Proliferation ofA549 and H2122 AGL knockdown (shAGL) cells following treatment with 4MU and 4MU+HA. Cells were plated in 96 well plate (10^3^ cells/well) (n=6). Next day cells were treated with vehicle control (PBS) of 4MU (600μM) or 4MU(600μM)+HA (20μg/ml). Proliferation was measured over a 5 day period by CyQUANT assay. ^*^p<0.05 by Student's t-test.

After establishing that the AGL knockdown cells synthesize more HA, we subjected the AGL knockdown H2122 and A549 cells to treatment with 4MU, an inhibitor of HA synthesis [11]. Treatment with increasing concentrations of 4MU resulted in decreased HA synthesis by these cells with maximum inhibition being achieved at a concentration of 600μM 4MU (Figure 5A, 5B). Next, we evaluated the effect of 4MU on proliferation of these AGL knockdown NSCLC cells. We treated H2122 and A549 AGL knockdown cells with 4MU at a concentration of 600μM in addition, we also treated cells with 4MU (600μM) plus low molecular weight HA (20μg/ml). We observe a decrease in proliferation of the AGL knockdown cells following treatment with 4MU which was partially rescued by addition of low molecular weight HA (Figure 5C, 5D). These results further validated that NSCLC cells become dependent on HA synthesis and signaling for growth with AGL loss and the reduction in proliferation seen with 4MU treatment is specific to reduction of HA synthesis by the cells.

### HA receptor RHAMM plays an important role in the aggressive growth of NSCLC cells that have lost AGL

**Figure 6:**
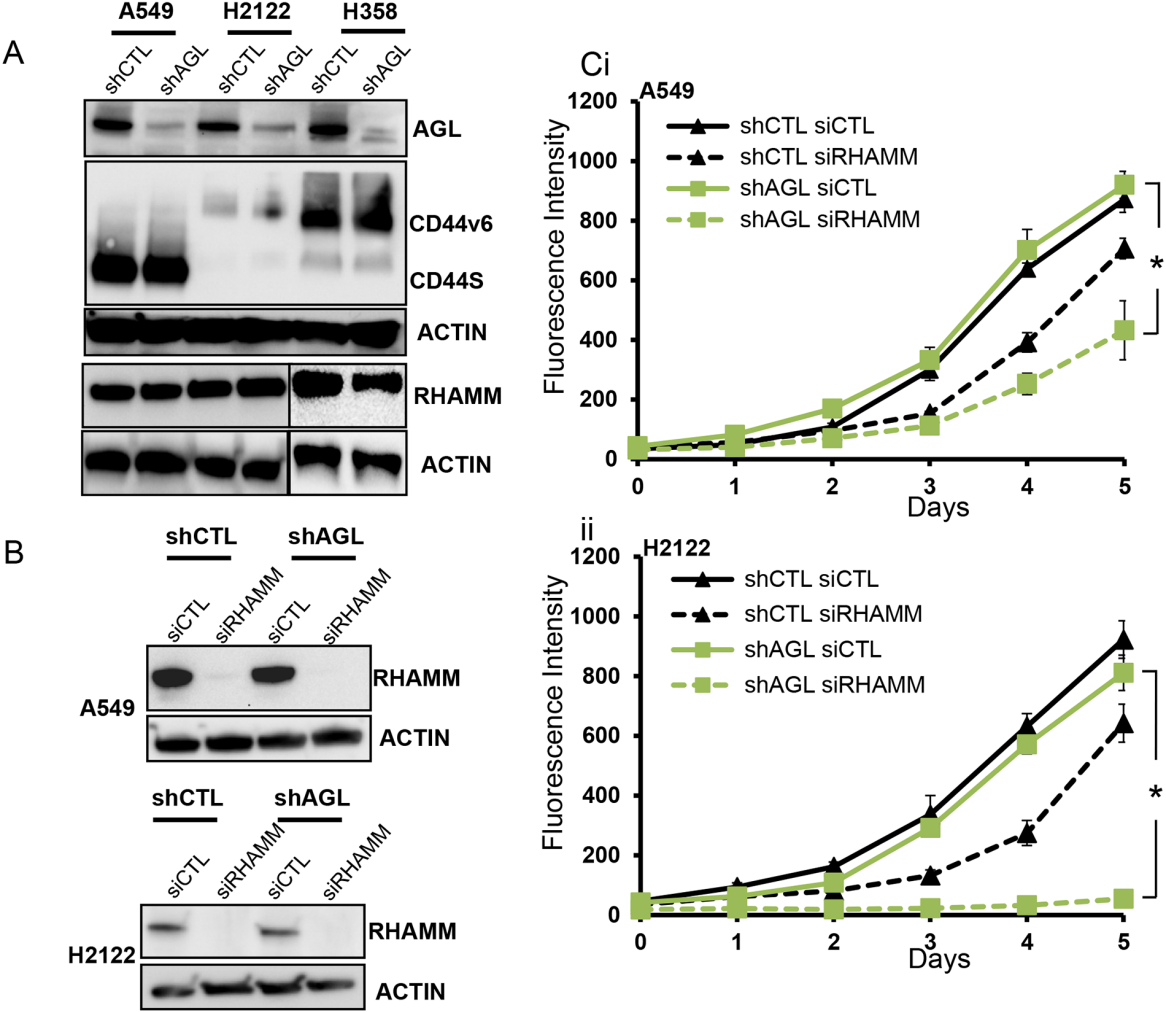
HA receptors and AGL loss in NSCLC **(A)** Expression of CD44 and RHAMM in NSCLC cells with (shCTL) or without (shAGL) AGL expression detected by Western blot (n=3). **(B)** Western blot demonstrating efficacy of RHAMM depletion in A549 and H2122 control (shCTL) and AGL knockdown (shAGL) cells. Cells were plated and 24hrs later transfected with scrambled (siCTL) or directed siRNA against RHAMM (siRHAMM). Details of siRNA used are in Materials and Methods. Cells were harvested at 72hrs for protein followed by Western blot (n=3). **(Ci-ii)** Proliferation of A549 and H2122 control (shCTL) and AGL knockdown (shAGL) cells after depletion of RHAMM. Cells were plated and 24hrs later transfected with scrambled (siCTL) or directed siRNA against RHAMM (siRHAMM). 48 hrs after transfection cells were plated in 96 well dish (10^3^ cells/well) (n=6) for proliferation over 5 days. Cell proliferation was measured by CyQUANT assay. Results are shown as mean±SD, ^*^p<0.05 by Student's t-test.

To address the role of the two major HA receptors CD44 and RHAMM [12, 13] in the three NSCLC cell lines we looked at expression of these receptors with and without AGL loss. CD44 standard isoform (CD44S) is expressed only by A549 cells; H2122 have low expression of CD44 and H358 express the v6 variant of CD44 (CD44v6) (Figure 6A). Expression of CD44 does not change with knockdown of AGL (Figure 6A). The other HA receptor, RHAMM was expressed equally by all the cell lines and the expression was unaffected by changes in AGL levels (Figure 6A). Since H2122 do not express CD44 and a previous study has shown that in H358 cells CD44 do not interact with HA [14], we ruled out CD44 as an important receptor for HA signaling in these cell lines. We hypothesized that RHAMM might be important for HA signaling in these NSCLC cells and with AGL loss these cells might be more dependent of RHAMM induced signaling for growth.

We carried out RHAMM knockdown with a previously validated siRNA [5] in A549 and H2122 NSCLC cells with and without AGL loss (Figure 6B). Loss of RHAMM mainly inhibited the proliferation of the AGL knockdown NSCLC cells with H2122 AGL knockdown cells showing the greatest inhibition of cell growth following loss of RHAMM (Figure 6C). This data supports our hypothesis that RHAMM is important for growth of the AGL knockdown NSCLC cells.

### AGL in combination with HAS2 or RHAMM predict patient outcome in NSCLC

**Figure 7:**
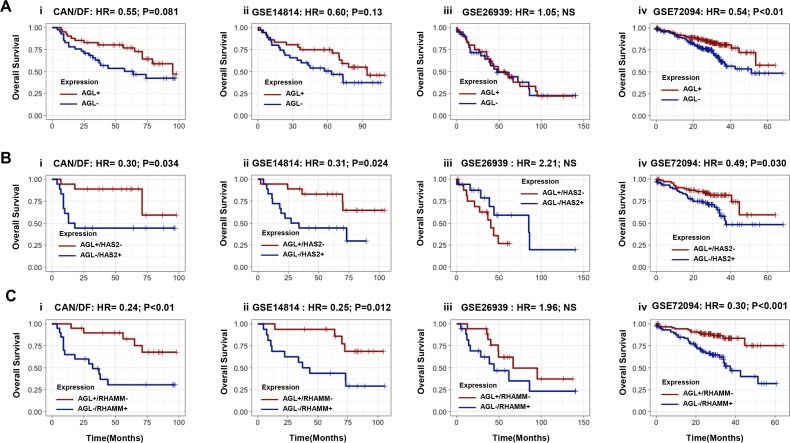
Relationship of AGL, HAS2 and RHAMM mRNA to predict NSCLC patient outcome **(Ai-iv)** Kaplan–Meier analysis of categorized median mRNA levels of AGL and overall survival in four independent NSCLC patient datasets. **(Bi-iv)** Kaplan–Meier analysis of categorized median mRNA levels of AGL and HAS2 (High AGL/Low HAS2 vs Low AGL/High HAS2) and overall survival in four independent NSCLC datasets. **(Ci-iv)** Kaplan–Meier analysis of categorized median mRNA levels of AGL and RHAMM (High AGL/Low RHAMM vs Low AGL/High RHAMM) and overall survival in four independent NSCLC datasets. Details of datasets are in Materials and Methods. Hazard Ratios (HR) and logrank P values are shown.

The role of AGL as a predictor of NSCLC patient outcome is not known. We analyzed 4 independent patient cohorts of NSCLC adenocarcinoma patients with Stage I and II tumors (N = 555) [15–18] to determine if AGL mRNA expression predicts patient outcome. The clinical characteristics of the patient cohorts were varied with respect to age, stage, and median follow up times (Supplementary Table 1). Segregating patients based of median AGL mRNA expression we show that in 3 of these 4 cohorts low AGL expression trend towards poor patient overall survival (Figure 7A) with one of these cohorts (Figure 7Aiv) showing statistically significant (P>0.05) association between AGL mRNA expression and patient outcome.

We have shown that HAS2 expression is elevated with loss of AGL. Next we investigated whether AGL and HAS2 in combination can predict patient outcome. The primary objective here was to determine if such expression levels could eventually be used to identify the optimal patient cohort who may be enrolled in future clinical trials with inhibitors of HA signaling. The secondary objective was to lend credence to the hypothesis that AGL affects tumor biology by HAS2 mediated HA synthesis. We segregated patients with high AGL and low HAS2 (AGL+/HAS2-) mRNA expression from patient with low AGL and high HAS2 mRNA expression (AGL-/HAS2+). Kaplan–Meier survival showed that patients with AGL-/HAS2+ have poor overall survival compared to AGL+/HAS2- patients (Figure 7B) with statistically significant P values in 3 of the 4 independent patient cohorts analyzed.

Similarly we looked at whether AGL and RHAMM, the HA receptor important for growth of AGL knockdown NSCLC cells, stratify NSCLC patient outcome. Kaplan–Meier survival show that patients with low AGL and high RHAMM mRNA expression (AGL-/RHAMM+) have poor overall survival compared to patients with high AGL and low RHAMM expression (AGL+/RHAMM-) with significant P values in 3 of the 4 patient cohorts analyzed (Figure 7C). Furthermore, the relationship between AGL/HAS2 and AGL/RHAMM expression and outcome was independent of stage in nearly all cases (Table 1). This data again lends credence to the hypothesis that AGL affects tumor growth by HAS2 mediated HA synthesis and signaling via RHAMM. This study will help identify the ideal NSCLC patients cohort based on AGL, HAS2 and RHAMM mRNA expression who would likely respond to inhibition of HA signaling.

**Table 1:** Multivariate survival analysis

	CAN/DF [15]	GSE14814 [18]	GSE26939 [17]	GSE72094 [16]
AGL+ vs AGL-	0.62 (0.168)	0.69 (0.288)	0.96 (0.892)	**0.56 (0.015)**
Stage II vs Stage I	**3.35 (<0.01)**	**2.14 (0.028)**	**2.15 (0.02)**	**2.05 (<0.01)**
AGL+/HAS2- vs AGL-/HAS2+	0.33 (0.066)	**0.32 (0.039)**	2.03 (0.203)	**0.51 (0.042)**
Stage II vs Stage I	**3.44 (0.029)**	**3.91 (0.013)**	1.69 (0.347)	**3.17 (0.001)**
AGL+/HMMR- vs AGL-/HMMR+	**0.32 (0.045)**	**0.28 (0.034)**	0.63 (0.346)	**0.33 (<0.01)**
Stage II vs Stage I	2.1 (0.154)	1.78 (0.293)	**3.8 (0.017)**	**2.35 (<0.01)**

Multivariate survival analyses were performed based on gene expression profiles and stage in each cohort. High (+) and low (-) expression is relative to the median expression value. Results are in the form Hazard Ratio (p-value).

## DISCUSSION

Here for the first time we show that glycogen debranching enzyme (AGL) regulates NSCLC tumor growth. We further validate that AGL regulates NSCLC growth independent of its enzymatic function which is consistent with our previous findings in the bladder tumor model [3]. Here we show for the first time that HAS2 driven HA synthesis and signaling through RHAMM is a major driver of growth in NSCLC cells that have lost AGL expression and provide preclinical evidence that personalized inhibition of the HAS2-HA-RHAMM axis may benefit patients with low tumor AGL and high HAS2 or RHAMM expression. Even though HA has been previously shown to be a driver of growth and metastasis in various tumor models including NSCLC [11–13, 19–23], here we show the importance of HA signaling in a subset of lung tumors which lack AGL expression.

We have earlier shown that in bladder tumors, loss of AGL promotes rapid anchorage dependent and independent growth of cancer cells [3]. However in bladder tumors, loss of AGL profoundly increased anchorage independent growth and xenograft growth [3]. In NSCLC we see that loss of AGL only results in increased anchorage independent growth compared to control cells. Thus loss of AGL primarily promotes the growth of these cells when they are not attached to a surface or when they don't need a surface to divide and spread. Anchorage independent growth is a measure of how oncogenic a cancer cell is and the potential of these cells to grow away from the site of its origin or be metastatic [24]. This proves that loss of AGL makes NSCLC cells more aggressive and oncogenic under the stressful condition of anchorage independence. This also suggests that loss of AGL might also promote metastasis of these cells which needs further investigation.

We show that with loss of AGL there is an increase in HAS2 expression and hyaluronic acid synthesis. It is well established that HAS2 driven HA synthesis or HA in general plays a major part in promoting anchorage dependent and independent growth [9, 10]. Inhibition of HA synthesis or HA signaling has been shown to inhibit anchorage independent and independent growth of cancer cells [25]. We believe that an increase in HAS2 driven HA synthesis is a major driver for increased anchorage independent growth of NSCLC cells expressing low levels of AGL. The fact that loss of AGL does not increase monolayer growth of NSCLC cells suggests that under favorable growth conditions increase in HA synthesis or signaling with knockdown of AGL do not provide added growth advantage to these cells compared to the control cells. However increased HA synthesis and signaling is vital for anchorage dependent monolayer growth of NSCLC cell lines with AGL loss, since loss of HAS2, RHAMM or treatment with HA synthesis inhibitor 4MU have a major inhibitory effect on the anchorage dependent monolayer growth of AGL knockdown NSCLC cells but not control cells. This validates that increased HA synthesis and signaling is important for both anchorage dependent and independent growth of NSCLC cells with AGL loss.

Another important aspect which merits discussion is HA size and their impact on tumor growth. Hyaluronic Acid Synthases (HAS1, HAS2 and HAS3) are known to make HA of different sizes [26]. HAS2 which is upregulated with AGL loss is known to make HA in the size range of 2000kDa or higher [26]. However HA synthesized constantly gets degraded by hyaluronidases and reactive oxygen species to smaller fragments or low molecular weight HA (20-40kDa) [26, 27]. These low molecular weight HA are known to bind to various cell surface receptors such as RHAMM and CD44 to induce oncogenic signaling [26, 27]. It is difficult to access the composition of different sized HA fragments which might be present in or secreted into the media by cells with and without AGL expression. Also it is extremely difficult to decipher which HA fragment might provide growth advantage to the AGL knockdown cells. We used commercially available low molecular weight HA (15-40kDa) to show it can partially rescue growth inhibition caused by 4MU in AGL knockdown NSCLC cells suggesting that HA synthesized by HAS2 gets degraded to that smaller size to induce its protumorigenic effect. However the partial rescue with exogenous HA use, which is consistent with other researchers [4, 19, 20], speaks to the point that HA made and processed by cells are unique to them and its effects cannot be replicated by commercially available HA. Detailed characterization of the HA pool present in cells and secreted into the media of cells with AGL loss will help understand which HA fragments are the major drivers of growth with AGL loss. Also the role of hyaluronidases in generating these HA fragments is extremely important to fully understand HA function with AGL loss.

It is still unclear how loss of AGL results in increased HAS2 expression and HA synthesis. Our previous study in the bladder tumor model indicated metabolic reprogramming and increased glucose uptake and glycolysis with AGL loss may play an important role in increasing HA synthesis [3, 4]. Glucose is the primary starting substrate for HA precursors [28]. High cytosolic glucose is known to activate PKC and increase HAS2 expression and HA synthesis [29, 30]. However it is still unclear how loss of AGL might result in metabolic reprogramming. It has been clearly shown by us here and in bladder cancer [3] that loss of AGL's enzymatic activity or inhibition of glycogen breakdown does not positively impact tumor growth. It needs to be investigated in detail how loss of AGL brings about metabolic reprogramming and whether increase in HAS2 expression and HA synthesis is a result of metabolic reprogramming or AGL's potential interaction with an unknown effector protein to directly regulate HAS2 expression. Currently mass spectrometric analysis is underway to decipher AGL-effector protein interactions to understand how loss of AGL might result in elevated HAS2 expression and aggressive tumor growth.

We have shown that AGL, in combination with HAS2 or RHAMM, predict NSCLC patient outcome. Patients with low AGL and high HAS2 or RHAMM expression had poor overall survival compared to patients with high AGL and low HAS2 or RHAMM expression. This provides credence to our findings that HA synthesis and signaling is important for tumors with low AGL expression. This data will also help in the selection of the ideal patient cohort for future intervention with inhibitors of HA synthesis and signaling.

4MU is a HA synthesis inhibitor which has been well studied in the field of cancer biology [11]. A great deal of preclinical data in various tumor models show promising results using 4MU to inhibit tumor growth and metastasis [3, 11, 21, 22, 31]. RHAMM inhibitory peptides have also been developed for treatment of cancer patients [32, 33]. A few clinical trials with these inhibitors have failed due to adverse effects on normal cells [32]. For an anti-HA treatment to be effective as a cancer therapy we believe an optimal patient cohort who might have the best response to this treatment strategy must be identified. Cancer patients where AGL expression is low and HAS2 or RHAMM is overexpressed might be the best patient cohort who would responds to HA synthesis or signaling inhibitors. We plan to test these therapeutic strategies on the bladder and NSCLC tumor model in the near future.

## MATERIALS AND METHODS

### Cell line and biochemical reagents

NSCLC cell lines H358, H2122 and A549 were obtained from the American Type Culture Collection (ATCC, Manassas, VA, USA) and cultured in RPMI-1640 (Invitrogen, Grand Island, NY, USA) supplemented with 10% fetal bovine serum (FBS) as recommended by ATCC. These three NSCLC cell lines were chosen for the study because they show an induction in growth with AGL loss; therefore serve as good model cell lines to study AGL biology in NSCLC. These cell lines were also chosen because they are known to form xenografts and hence will allow us to study AGL biology in *in vitro* and *in vivo* setting. Short hairpin RNA (shRNA) sequence 5'-CCGGCCCTTGCCAATCAGTTAGAATCTCGAGATTCTAACTGATTGGCAAGGGTTTTTG-3' (TRCN0000035082, Sigma-Aldrich) [3–5] was used for human AGL in lentiviral plasmid vector pLKO.1-Puro (Sigma) as previously used and shRNA sequence 5'-CCGGATATTAACACCACGTACTATACTCGAGTATAGTACGTGGTGTTAATATTTTTTTG-3' (TRCN0000419324, Sigma-Aldrich) targeting AGL 3'UTR region was also used as a second construct. shRNA sequences 5'-CCGGCACGAAGAAGACCTGTGCATACTCGAGTATGCACAGGTCTTCTTCGTGTTTTTTG-3' (TRCN0000158010, Sigma-Aldrich) was used for human glycogen phosphorylase brain (GYPB) isoform; shRNA sequences 5'-CCGGCCTCGACATTTGGAAATCATTCTCGAGAATGATTTCCAAATGTCGAGGTTTTTG-3'(TRCN0000119086, Sigma-Aldrich) was used for human glycogen phosphorylase liver (GYPL) isoform as previously used [3]. Human AGL construct (vectorEX-E2057-Lv102) was purchased from GeneCopoeia (Rockville, MD). AGL enzymatic mutants L620P and R1147G were made using site directed mutagenesis using mutagenesis primers: forward 5'- GCCAGCTATTGCACATGCCCCCTTTATGGATATTACG-3' reverse 5'- CGTAATATCCATAAAGGGGGCATGTGCAATAGCTGGC-3' and forward 5'- GTGAAGGAATTTATGCCGGCTACAATTGTCGGGATG-3' reverse 5'- CATCCCGACAATTGTAGCCGGCATAAATTCCTTCAC-3' respectively from IDT. 4-Methylumbelliferone (4-MU, cat. # M1508-10G) was obtained from Sigma-Aldrich. Low Molecular weight HA (cat. # GLR001) was obtained from R&D systems (Minneapolis, MN). Low molecular weight HA has been previously shown by us and others to be protumorigenic [4, 19, 26] hence have been used in this study. siRNA sequences 5'-GGTTTGTGATTCAGACACT-3' was used at a concentration of 50 nM to knockdown HAS2 (siHAS2) as previously reported [4, 5]. siGENOME SMARTpool siRNAs were used to RHAMM (M-010409-01-0005, siRHAMM) at a concentration of 20 nM [5] as previously reported. siRNA's were purchased from Dharmacon (Lafayette, CO, USA) and transfected using Lipofectamine RNAiMAX (Invitrogen) using manufacturer instructions. NSCLC cell lines were authenticated by the University of Colorado PPSR core using an Applied Biosystems Profiler Plus Kit which analyzed 9 STR loci (Life Technologies 4303326). After authentication cells were frozen within 1-2 weeks. Vials of cells were resuscitated less than 2 months prior to being used in experiments in this study.

### PCR and western blot

HAS1-3 mRNA expression was determined by the ΔΔCT method [3, 5] with GAPDH as control for NSCLC cell lines with and without AGL expression. Expression was normalized to control cells transfected with control siRNA to determine HAS2 gene expression and knockdown in control and AGL knockdown cells with HAS2 siRNA treatment. HAS1 primer: forward 5'-TGTGCTGCGTCTGTTCTAC-3' reverse 5'-CTCTGGTTCATGGTGACTAGC-3'; HAS 2 primer: forward 5'-TCCCGGTGAGACAGATGAGT-3' reverse 5'- GGCTGGGTCAAGCATAGTGT-3'; HAS3 primer: forward 5'-TCCCTCTACTCCCTCCTCTAT-3' reverse 5'-CTGAACAGGTCCTGGCAATAA-3'; GAPDH primer: forward 5'-TCTTTTGCGTCGCCAGCCGA 3' reverse 5'- ACCAGGCGCCCAATACGACC-3' were used for the RT-PCR experiments as previously used [4].

Antibodies used for westerns were anti-AGL (Agrisera, Vannas, Sweden), Actin (GeneTex, Irvine, CA, USA), CD44 (Cell Signaling), RHAMM (Abcam, Cambridge, MA, USA). HRP (Cell Signaling) labeled mouse or rabbit secondary antibodies were used for chemiluminescence detection with ECL reagents (Pierce, Rockford, IL, USA) as previously described [3–5].

### Anchorage independent and dependent growth

Anchorage dependent and independent proliferation was measured as previously described [3, 4, 34]. Anchorage-independent growth was assessed by plating cells in 0.4% agar in triplicate. Briefly, H358, H2122 and A549 cells with or without AGL expression were plated (15,000 cells/well) in triplicate in 6 welled dish. Colonies were stained with Nitro-BT (Sigma) at the end of the experiment and counted using Image J.

For anchorage dependent growth assay, cells with or without AGL expression were transfected with control siRNA or siRNA targeting HAS2 or RHAMM [5]. 72hrs after transfection cell proliferation and viability was assessed by plating 10^3^ cells per well in 96-well plates in triplicate for proliferation studies. CyQUANT® Cell Proliferation Assay (Invitrogen) was carried out according to manufacturer instructions to measure cell proliferation. To determine the effects of 4MU on cell viability, cells were plated as described, and treated with 4MU (600 μM) or 4MU (600μM) and HA(20μg/ml) together for 5 days. Cell viability was determined by CyQUANT Assay (Invitrogen).

### HA ELISA

Fresh media is applied 48hrs after HAS2 siRNA transfection in AGL knockdown cells followed by HA analysis by ELISA 24 hrs later. Cells with and without AGL are grown to 60-65% confluency followed by fresh media addition with increasing concentrations of 4MU to evaluate the impact of 4MU on HA synthesis and secretion after 24hrs. HA ELISA was conducted as per manufacturer instructions using TECO® HA ELISA kit.

### Xenograft assay

All animals used in this study were treated according to Institutional Animal Care and Use Committee (IACUC) guidelines. Four-week-old NCr nu/nu female mice from Charles Rivers or Envigo were injected with H358 (4×10^6^), H2122 (1.0×10^5^) or A549 (2×10^6^) cells stably expressing AGL shRNA or nontarget shRNA control (7 mice per group) in the right and left flanks of each mouse for subcutaneous tumor growth. Tumors were measured and tumor volumes calculated as described previously [35]. In brief, the length (L) and width (W) of each tumor were measured using calipers, and tumor volume was calculated using the equation (L × W^2^)/2. Animals without tumor take (i.e. measurement of 0) were excluded from tumor volume calculations.

### Bioinformatics and statistical analyses

CEL files for the CAN/DF cohort were downloaded [15] and processed using the robust multiarray average (RMA) method [36]. Processed gene expression data was downloaded from the Gene Expression Omnibus, Accession #GSE14814 [18], GSE26939 [17], GSE72094 [16]. Analysis of variance (ANOVA) and Fisher's exact test were used to determine if group means or proportions differed across the patient cohorts. For survival analysis, expression levels were categorized as high (+) if they exceeded the median expression value across all samples, or low (-) otherwise. Kaplan–Meier curves were generated for patients based on low and high expression values, with a hazard ratio (HR) and log-rank P-value reported. Analyses were carried out using cox proportional hazard models (*survival* package) in *R* (https://cran.r-project.org/).

## SUPPLEMENTARY MATERIALS FIGURES AND TABLE



## Abbreviations

NSCLC: non-small cell lung cancer
SHMT2: Serine Hydroxymethyltransferase 2
HAS2: Hyaluronic acid synthase 2
HA: Hyaluronic acid
4MU: 4-methylumbelliferone
ELISA: Enzyme-linked immunosorbent assay
RHAMM: Hyaluronan mediated motility receptor
GSDIII: Glycogen storage disease III
