# Supplementary material for: Glycogen debranching enzyme (AGL) is a novel regulator of non-small cell lung cancer growth
Source: Oncotarget. 2018 Mar 30;9(24):16718–30. doi: 10.18632/oncotarget.24676 (PMC5908281; doi:10.18632/oncotarget.24676)
Supplement: Supplementary file 1 [file oncotarget-09-16718-s001.pdf]

Glycogen debranching enzyme (AGL) is a novel regulator of non-small cell lung cancer growth

SUPPLEMENTARY MATERIALS

REFERENCES

1. Shedden K, Taylor JM, Enkemann SA, Tsao MS, Yeatman TJ, Gerald WL, Eschrich S, Jurisica I, Giordano TJ, Misek DE, Chang AC, Zhu CQ, Strumpf D, et al, and Director's Challenge Consortium for the Molecular Classification of Lung Adenocarcinoma. Gene expression-based survival prediction in lung adenocarcinoma: a multi-site, blinded validation study. Nat Med. 2008; 14:822–27. https://doi.org/10.1038/nm.1790cre.
2. Zhu CQ, Ding K, Strumpf D, Weir BA, Meyerson M, Pennell N, Thomas RK, Naoki K, Ladd-Acosta C, Liu N, Pintilie M, Der S, Seymour L, et al. Prognostic and predictive gene signature for adjuvant chemotherapy in resected non-small-cell lung cancer. J Clin Oncol. 2010; 28:4417-24. https://doi.org/10.1200/JCO.2009.26.4325.
3. Wilkerson MD, Yin X, Walter V, Zhao N, Cabanski CR, Hayward MC, Miller CR, Socinski MA, Parsons AM, Thorne LB, Haithcock BE, Veeramachaneni NK, Funkhouser WK, et al. Differential pathogenesis of lung adenocarcinoma subtypes involving sequence mutations, copy number, chromosomal instability, and methylation. PLoS One. 2012; 7:e36530. https://doi.org/10.1371/journal.pone.0036530.
4. Schabath MB, Welsh EA, Fulp WJ, Chen L, Teer JK, Thompson ZJ, Engel BE, Xie M, Berglund AE, Creelan BC, Antonia SJ, Gray JE, Eschrich SA, et al. Differential association of STK11 and TP53 with KRAS mutation-associated gene expression, proliferation and immune surveillance in lung adenocarcinoma. Oncogene. 2016; 35:3209-16. https://doi.org/10.1038/onc.2015.375.

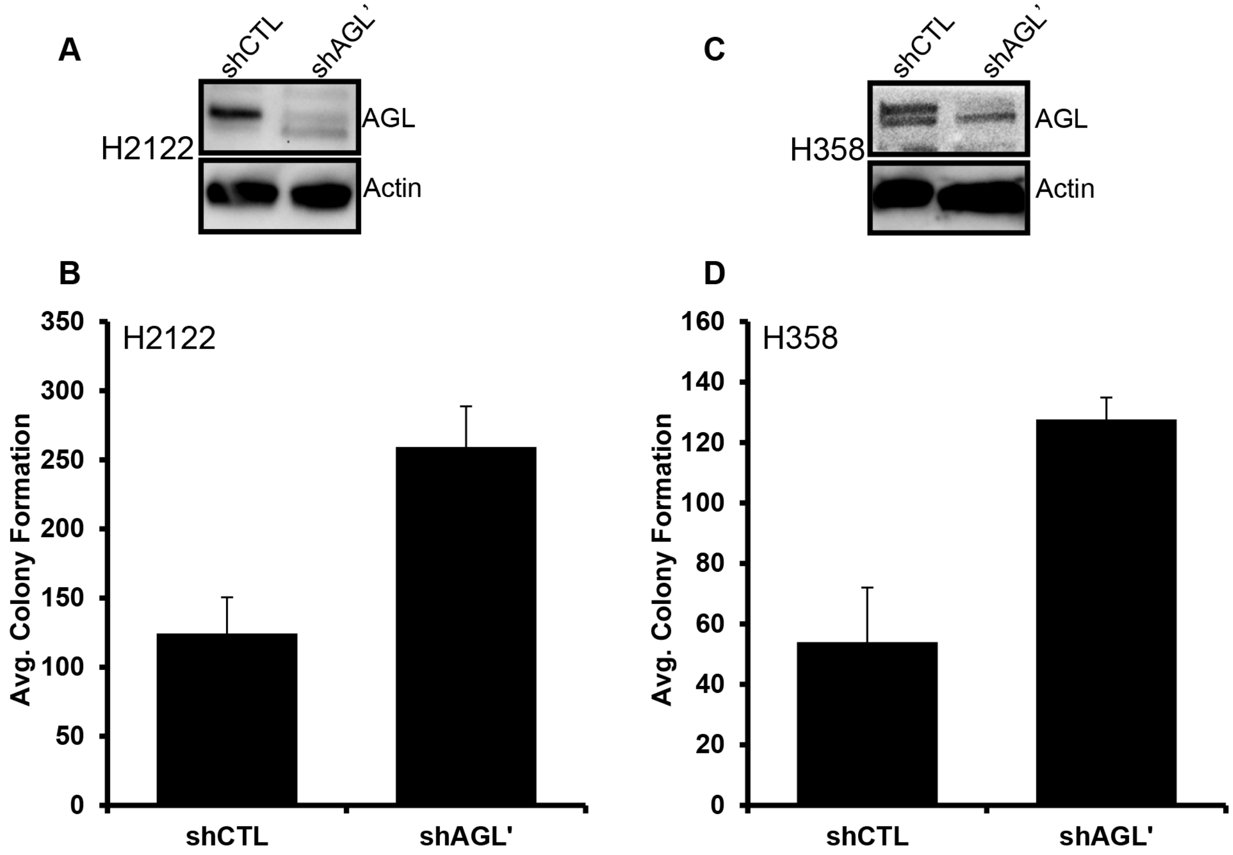

Supplementary Figure 1: (A, C) AGL gene knockdown was validated by Western blot in the NSCLC cell lines. Cells transduced with control shRNA is labeled as shCTL and cells transduced with AGL specific shRNA targeted at the 3'UTR region is labeled shAGL'. Details of the construct is in Materials and Methods. **(B, D)** Anchorage independent growth (n=3) of NSCLC cells with (shCTL) and without AGL (shAGL'; shRNA directed at 3'UTR region) expression. 15×10^3 cells were plated in 6 well plate for agar growth. Results are shown as mean±SD, $^*p<0.05$ by Student's t-test.

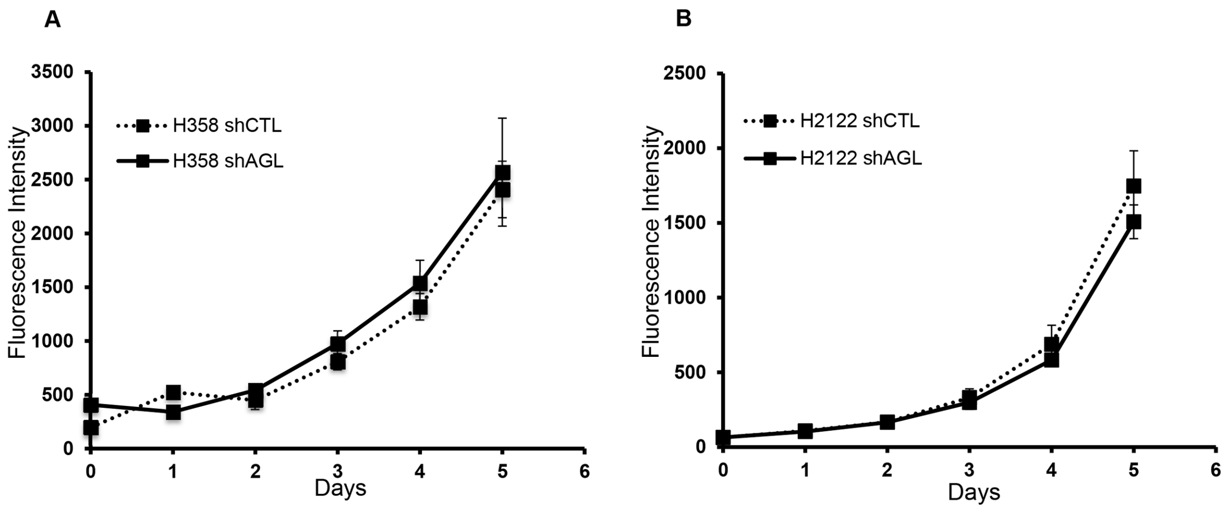

Supplementary Figure 2: (A, B) Proliferation of H358 and H2122 control (shCTL) and AGL knockdown (shAGL) cells. Cells were plated in 96 welled dish (10^3 cells/well) (n=6) for proliferation over 5 days. Cell proliferation was measured by CyQUANT assay. Results are shown as mean±SD, $^*p<0.05$ by Student's t-test.

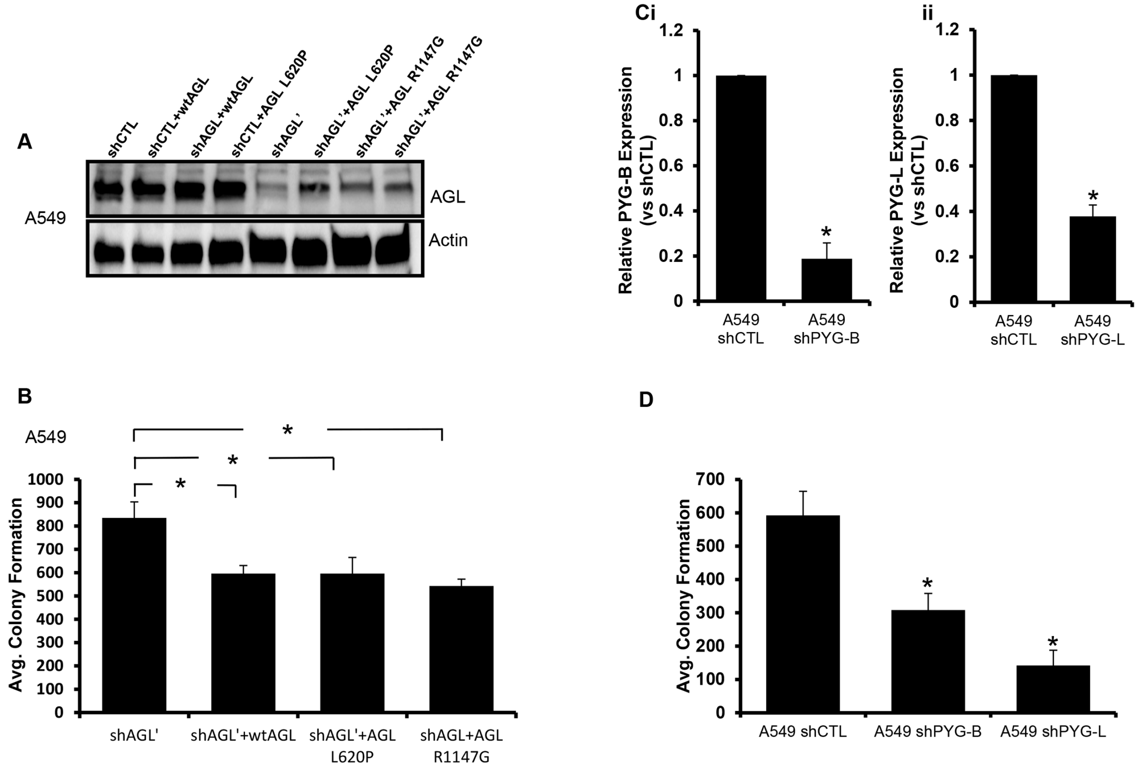

Supplementary Figure 3: **(A)** AGL expression in A549 cells transduced with nontarget shRNA (shCTL) and cells transduced with shRNA against AGL specific to 3'UTR region (shAGL') stably overexpressing WT-AGL and enzymatic null AGL. **(B)** Anchorage independent growth (n=3) of H2122 cells with (shCTL) and without AGL (shAGL') expression stably overexpressing WT-AGL and enzymatic null AGL. 15×10^3 cells per cell type were plated in 6 well plates for soft agar growth. **(Ci-ii)** qRT-PCR demonstrating efficacy of glycogen phosphorylase brain (shPYG-B) and liver (shPYG-L) isoform depletion in A549 cells stably transduced with shRNA against glycogen phosphorylase brain and liver isoform. **(D)** Anchorage independent (n=3) growth of A549 cells transduced with nontargeted shRNA and shRNA against glycogen phosphorylase liver (shPYG-B) and brain (shPYG-L) isoform. 15×10^3 cells were plated in 6 well plates for soft agar growth. Results are shown as mean±SD, $^*p<0.05$ by Student's t-test.

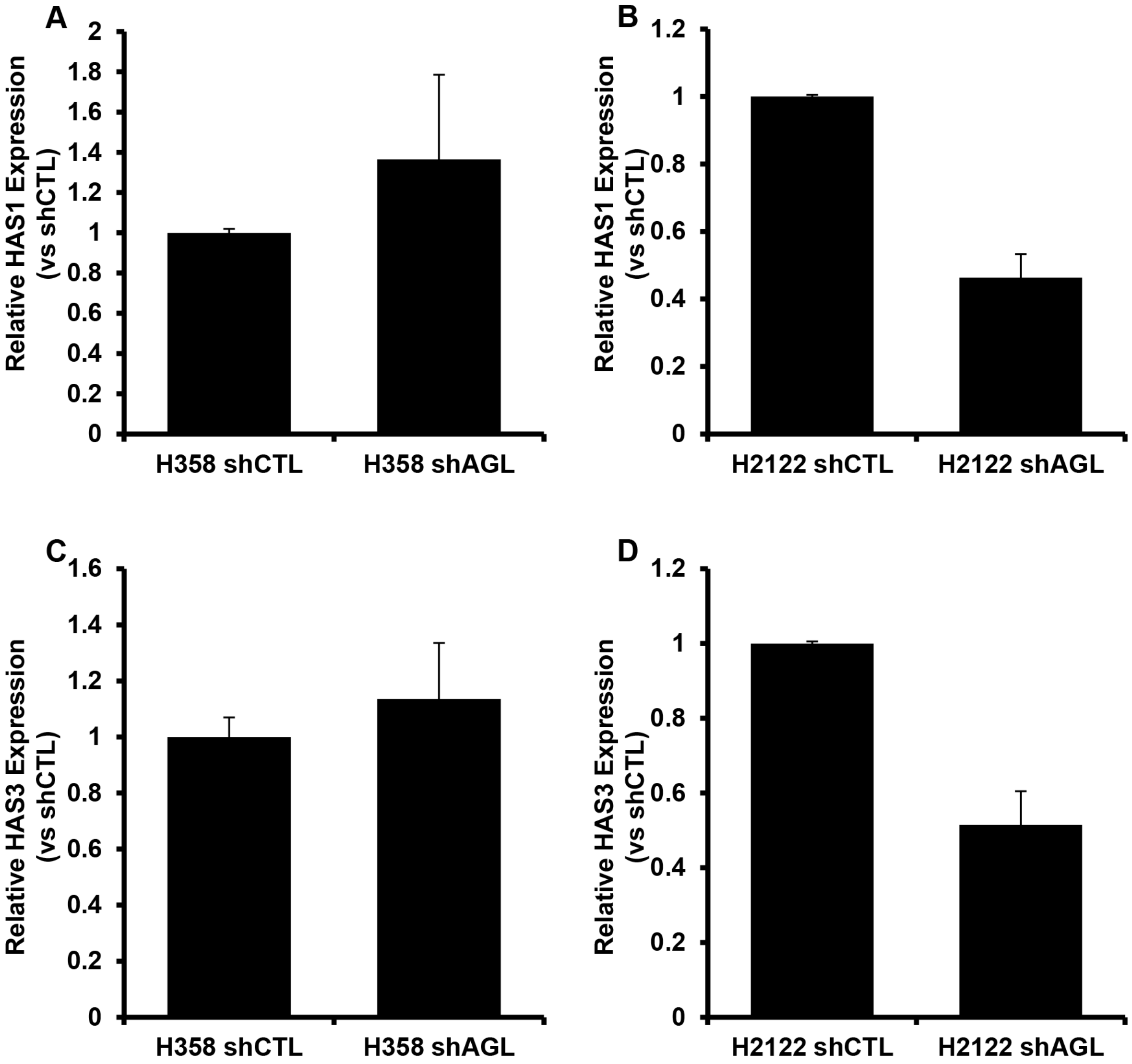

Supplementary Figure 4: **(A, B)** qRT-PCR demonstrating the expression of HAS1 in NSCLC cells H358 and H2122 with (shCTL) and without (shAGL) AGL expression (n=3). **(C, D)** qRT-PCR demonstrating the expression of HAS3 in NSCLC cells H358 and H2122 with (shCTL) and without (shAGL) AGL expression (n=3). Results are shown as mean±SD, $^{*}p<0.05$ by Student's t-test.

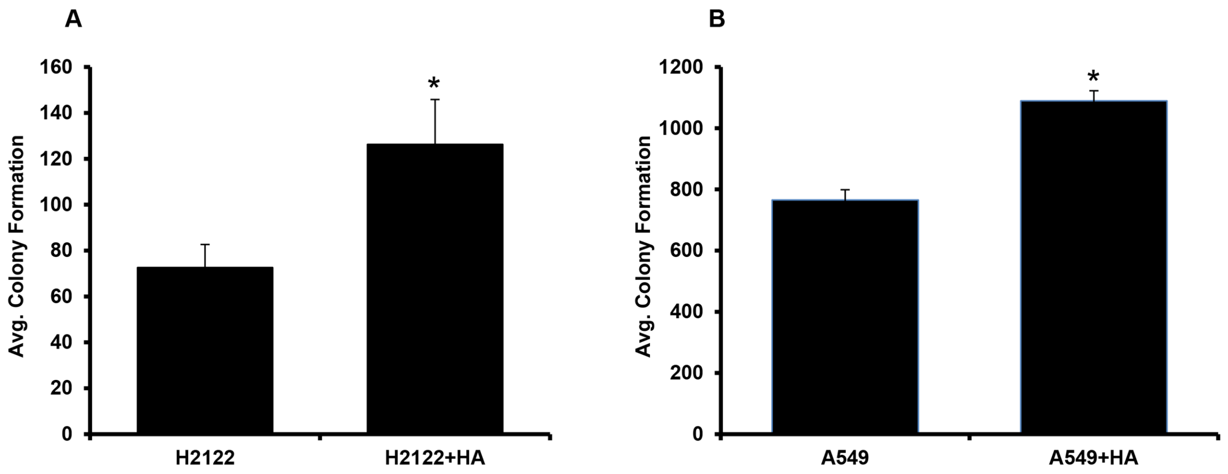

Supplementary Figure 5: **(A, B)** Anchorage independent growth (n=3) of NSCLC cells H2122 and A549 on treatment with HA. 15×10^3 cells were plated in 6 well plate for soft agar growth with and without HA (20µg/ml). $p<0.05$ by Student's t-test.

Supplementary Table 1: Clinical characteristics of patients

	CAN/DF [1]	GSE14184*[2]	GSE26939 [3]	GSE72094 [4]	P-value
Sample Size	82	71	81	321	
Mean Age (sd)	61 (10)	59 (9)	65 (11)	69 (9)	< 0.01
%Male (%Female)	56% (44%)	52% (48%)	44% (56%)	56% (44%)	0.29
Stage (I, II) (%)	(68%, 32%)	(59%, 41%)	(77%, 23%)	(79%, 21%)	0.003
Median Follow-up (months)	51	60	37	28	< 0.01
Number of deaths (%)	35 (43%)	35 (49%)	45 (56%)	90 (28%)	<0.01

* 55% of patients are treated with adjuvant cisplatin/vinorelbine.
